# The Evolutionary Landscape of Treatment for *BRAF^V600E^* Mutant Metastatic Colorectal Cancer

**DOI:** 10.3390/cancers13010137

**Published:** 2021-01-04

**Authors:** Gianluca Mauri, Erica Bonazzina, Alessio Amatu, Federica Tosi, Katia Bencardino, Viviana Gori, Daniela Massihnia, Tiziana Cipani, Francesco Spina, Silvia Ghezzi, Salvatore Siena, Andrea Sartore-Bianchi

**Affiliations:** 1 Niguarda Cancer Center, Grande Ospedale Metropolitano Niguarda, 20162 Milano, Italy; gianluca.mauri@ospedaleniguarda.it (G.M.); ericafrancesca.bonazzina@ospedaleniguarda.it (E.B.); alessio.amatu@ospedaleniguarda.it (A.A.); federica.tosi@ospedaleniguarda.it (F.T.); katiabruna.bencardino@ospedaleniguarda.it (K.B.); viviana.gori@ospedaleniguarda.it (V.G.); daniela.massihnia@ospedaleniguarda.it (D.M.); tiziana.cipani@ospedaleniguarda.it (T.C.); francesco.spina@ospedaleniguarda.it (F.S.); silvia.ghezzi@ospedaleniguarda.it (S.G.); salvatore.siena@unimi.it (S.S.); 2 Dipartimento di Oncologia ed Emato-Oncologia, Università degli Studi di Milano, 20122 Milano, Italy

**Keywords:** *BRAF*, colon cancer, immune checkpoint inhibitors, targeted agents, FOLFOXIRI

## Abstract

**Simple Summary:**

The *BRAF^V600E^* mutation accounts for 8–10% of metastatic colorectal cancer (mCRC) patients and it is an established prognostic factor. Median overall survival of this subset of patients is indeed so poor that it is similar to first line PFS of patients without this molecular alteration. An exception is represented by patients displaying concomitant MSI-H status who can benefit from immunotherapy with checkpoint inhibitors (CPIs). Recently, a targeted therapy with the combination of encorafenib and cetuximab provided for the first time a survival gain and thus translation in the clinic, even though acquired resistance limits the possibility of more than an incremental benefit. Many studies exploiting other different strategies are ongoing. In this review we present current therapies specifically headed to *BRAF^V600E^* mutant mCRC and systematically review ongoing clinical trials identifying different approaches under investigations: targeting MAPK pathway (monotherapy or combinations), targeting MAPK pathway combined with cytotoxic agents, intensive cytotoxic regimen combinations, targeted agents combined with CPIs, oxidative stress induction, and cytotoxic agents combined with antiangiogenic drugs and CPIs.

**Abstract:**

The *BRAF^V600E^* mutation is found in 8–10% of metastatic colorectal cancer (mCRC) patients and it is recognized as a poor prognostic factor with a median overall survival inferior to 20 months. At present, besides immune checkpoint inhibitors (CPIs) for those tumors with concomitant MSI-H status, recommended treatment options include cytotoxic chemotherapy + anti-VEGF in the first line setting, and a combination of EGFR and a BRAF inhibitor (cetuximab plus encorafenib) in second line. However, even with the latter targeted approach, acquired resistance limits the possibility of more than an incremental benefit and survival is still dismal. In this review, we discuss current treatment options for this subset of patients and perform a systematic review of ongoing clinical trials. Overall, we identified six emerging strategies: targeting MAPK pathway (monotherapy or combinations), targeting MAPK pathway combined with cytotoxic agents, intensive cytotoxic regimen combinations, targeted agents combined with CPIs, oxidative stress induction, and cytotoxic agents combined with antiangiogenic drugs and CPIs. In the future, the integration of new therapeutic strategies targeting key players in the *BRAF^V600E^* oncogenic pathways with current treatment approach based on cytotoxic chemotherapy and surgery is likely to redefine the treatment landscape of these CRC patients.

## 1. Introduction

Colorectal cancer (CRC) is the third most common diagnosed type of cancer and the third cause of cancer related death worldwide in both women and men [1]. Despite recent improvements in CRC treatment, only 12% of patients diagnosed with metastatic colorectal cancer (mCRC) are still alive after five years [2]. As per clinical guidelines, pan-*RAS*, *BRAF*, HER2, and mismatch repair (MMR) status assessments are recommended to define patients prognosis and treatment strategy [3,4,5]. Particularly, *BRAF* mutations account for 8–10% of mCRCs and more than 90% are missense mutations occurring in codon 600, leading to an aminoacidic substitution of a valine for a glutamic acid (V600E) [6]. Furthermore, *BRAF* mutations different from *^V600E^* (*BRAF^non-V600E^*) account for about 2% of mCRCs and they have been associated with specific clinicopathological features and a better clinical outcome [7,8,9,10,11]. Considering that *BRAF^-V600E^* mutation in mCRC is still a clinical unmet need, we focused our manuscript on treatment of *BRAF^V600E^* mutant mCRC.

In mCRC *BRAF^V600E^* mutation represents a poor prognostic factor and median overall survival (OS) of patients diagnosed with advanced disease harboring this mutation ranges between 10 to 20 months [7,12]. Biologically, *BRAF^V600E^* mutant mCRCs are frequently characterized by hypermethylation, microsatellite instability (MSI) and consensus molecular subtype 1 (CMS1) [13]. Particularly, MSI features and *BRAF^V600E^* mutations frequently overlap and up to 50% of *BRAF^V600E^* mutant mCRCs are also MSI [14,15,16]. Notably, those MSI mCRCs harboring *BRAF^V600E^* mutation are always sporadic and do not arise in the context of Lynch Syndrome [14,15,16]. This is relevant since MSI and microsatellite stable (MSS) mCRCs are well-known to represent two distinct diseases with specific etiology, prognosis and different treatment implications [13,17].

**Figure 1:**
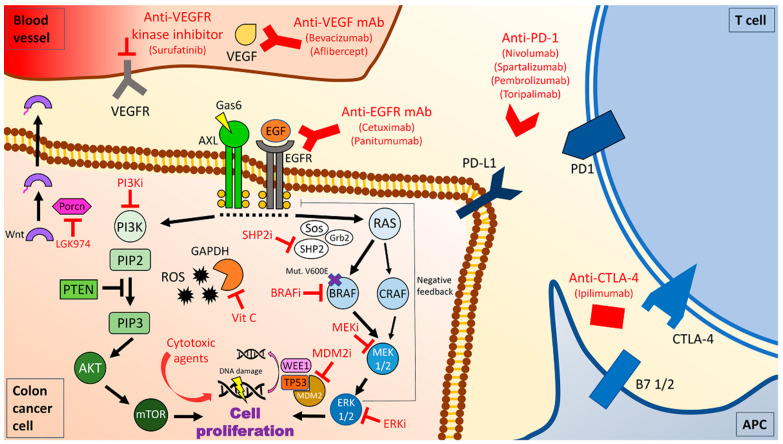
Schematic representation of pathways currently under investigations as actionable therapeutic targets harnessing *BRAF^V600E^* mutant metastatic colorectal cancer (mCRC). Legend: mAb = monoclonal antibodies; i = inhibitor. Vit = vitamin. Mut = mutation.

BRAF is a serine-threonine kinase playing a key role as downstream RAS effector in the mitogen-activated protein kinase (MAPK)/extracellular signal-regulated kinase (ERK) signal transduction cascade. *BRAF^V600E^* mutation causes an inappropriate activation of this pathway leading to uncontrolled cell proliferation, migration, angiogenesis, and escape from apoptosis [18] (Figure 1). *BRAF^V600E^* mutation is a target of treatment in various types of malignancies such as melanoma, non-small cell lung cancer (NSCLC), and hairy-cell leukemia [3,19,20,21].

In mCRC, initial studies targeting *BRAF^V600E^* mutant disease were disappointing, still demonstrating signs of activity [18,22,23]. However, initial exploitation of BRAF inhibitors as monotherapy in mCRC paved the way for the understanding of molecular mechanisms which led to rational combinations of MAPK targeting agents against *BRAF^V600E^* mutant disease [24,25,26,27]. Progressively, subsequent clinical trials reshaped the therapeutic landscape toward specific targeted or cytotoxic treatment regimens for this subset of patients [25,28]. However, prognosis of *BRAF^V600E^* mutant mCRC patients remains poor [12,18,25]. Further treatment improvements are needed to tackle this clinical still unmet need.

In this review, we first discuss current treatments options for *BRAF^V600E^* mutant mCRC patients and then we systematically review ongoing clinical trials focusing on novel strategies under investigation in this subset of patients.

## 2. Current Treatment Strategies 

**Figure 2:**
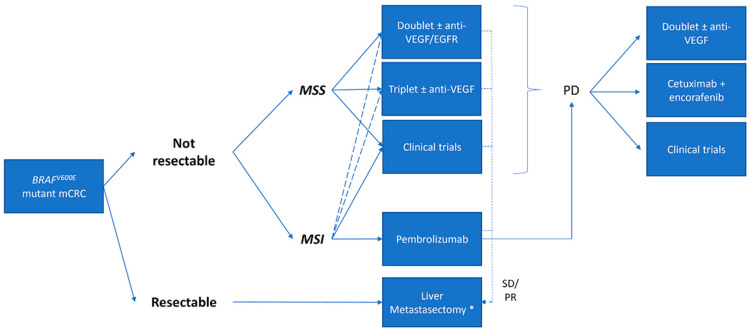
Current treatment options for *BRAF^V600E^* mutant metastatic colorectal cancer (mCRC). According to most recent studies, treatment opportunities for *BRAF^V600E^* mutant mCRC are fast developing if compared to only a decade ago. The panorama of treatment now includes the following options: surgery, combinations of cytotoxic drugs, targeted and immunological agents. All these approaches should be carefully evaluated when discussing the treatment approach to *BRAF^V600E^* mCRCs in multidisciplinary teams (MDT). Given the peculiarity of this subset of mCRCs, clinical trial enrolment should always be considered also in the upfront setting. Based on current evidence, MSI *BRAF^V600E^* mutant mCRC progressing to first line treatment with pembrolizumab should be managed as microsatellite stable (MSS) *BRAF^V600E^* mutant mCRC. Keys: * = Metastasectomy should be -considered in liver limited disease in case of response or prolonged disease control obtained with medical treatments even if relapse-free and overall survival is poorer than *BRAF* wild-type mCRCs. Legend: mCRC = metastatic colorectal cancer. SD = stable disease; PD = progressive disease; PR = partial response. “Dashed line” means consider. “Continuous line” means recommended.

Treatment strategies for *BRAF^V600E^* mutant mCRC have been the same of all mCRCs up to recent times. However, given the poor prognosis of these subset of patients, specific treatment regimens have been recently investigated with successful results [25,28]. These studies led to National Comprehensive Cancer Network (NCCN) and European Society of Medical Oncology (ESMO) recommendations of focused treatments improving outcomes [3,4,5]. Current options of treatment for *BRAF^V600E^* mutant mCRC are summarized in Figure 2. Although rarely *BRAF^V600E^* mutant mCRC patients present with liver or lung limited disease, international clinical guidelines recommend evaluating feasibility of surgical resection with curative intent in oligometastatic disease given its long-term survival implication [29,30]. However, it should be taken into account that shorter (OS) and relapse-free survival after metastasectomy have been reported [30,31,32]. In this regard, *BRAF^V600E^* mutation has been indicated as an exclusion criteria for most ongoing experimental trials of liver transplantation for mCRC, such as in the ongoing COLT Study (NCT03803436).

### 2.1. Immune Checkpoint Inhibitors in BRAF^V600E^ Mutant MSI-H mCRC

A fundamental step to identify the best treatment for a *BRAF^V600E^* mutant mCRC patient, given the increasing number of evidences showing a dramatic impact of treating MSI mCRC with checkpoint inhibitors (CPIs) [33,34,35,36], is the assessment of tumor’s MMR status. In the CheckMate 142 trial, 12 out of 74 MSI mCRC patients treated with nivolumab had *BRAF^V600E^* mutant disease [36]. Overall response rate (ORR) and disease control rate (DCR) were 31 and 69% in BRAF wild-type mCRCs and 25 and 75% in *BRAF^V600E^* mutant mCRCs [36]. In the nivolumab and ipilimumab cohort 29 out of 119 patients had *BRAF^V600E^* mutant MSI mCRC [33]. In this cohort response rates were higher in both *BRAF^V600E^* mutant and wild-type mCRC with a remarkable 55% ORR and 80% DCR in the former group [33]. In addition, the recent phase III trial KEYNOTE-177 demonstrated the superiority of pembrolizumab in first-line setting over standard regimens in MSI mCRC, independently from *BRAF* status (Table S1) [35]. According to these trials, CPIs seem to perform better than standard therapies in *BRAF^V600E^* mutant MSI mCRC [25,33,35]. The ongoing phase III trial CheckMate 8HW (NCT04008030) is evaluating the combination of nivolumab and ipilimumab in the same setting and it is expected to provide further data for this subset of patients. Summarizing, these studies support the administration of a CPI as upfront treatment in *BRAF^V600E^* mutant MSI mCRC patients. Indeed, following KEYNOTE-177 data, both Food and Drug Administration (FDA) and European Medicines Agency (EMA) recently approved pembrolizumab in the first line setting for MSI mCRC, including those *BRAF^V600E^* mutant [37,38]. Today, pembrolizumab is the new standard of care for MSI mCRC harboring *BRAF^V600E^* mutation. If immunotherapy is contraindicated or not available, standard cytotoxic treatments remain an option (Figure 2).

### 2.2. Doublet Cytotoxic Combination Plus Biological Agents

Standard doublet chemotherapy leads to poor outcome in terms of progression-free survival (PFS) in advanced mCRC harboring *BRAF^V600E^* mutation in first-, second- and third-line treatment [39]. Furthermore, the use of oxaliplatin or irinotecan does not modify PFS to first-line treatment [39].

In addition to standard cytotoxic agents, the added value of an anti-VEGF drug has never been shown through a dedicated trial in mCRCs harboring *BRAF^V600E^* mutation. However, AVF2107g and AGITG MAX trials showed a numerical improvement in survival outcomes for patients with *BRAF^V600E^* mutant mCRC with the addiction of bevacizumab to cytotoxic agents [40,41]. Also, a subgroup analysis of the second-line study VELOUR described a greater benefit in terms of OS from the addition of aflibercept to FOLFIRI in *BRAF^V600E^* mutant mCRC patients than in the wild-type ones, even though by its nature it is not powered to drive conclusions for this subset of patients [42].

As far as anti-EGFR treatment, initial data generated retrospectively in the advanced lines with cetuximab or panitumumab used as monotherapy, and supported by in vitro data, clearly showed that *BRAF^V600E^* mutation is a mechanism of resistance to this treatment [43]. This hypothesis has then been tested in subgroup analyses of prospective trials with conflicting results. In second line treatment, the addition of anti-EGFR to FOLFIRI did not confer any clinical benefit in *BRAF^V600E^* mutant mCRC patients and it is reported as potentially deleterious [44,45]. In contrast, in a first line setting, *BRAF^V600E^* mutation was not identified as a negative predictive biomarker of response to cetuximab or panitumumab added on top to FOLFOX or FOLFIRI, but rather a poor prognostic biomarker [46,47]. To assess the real impact of *BRAF^V600E^* mutation as predictive biomarker to anti-EGFR treatment, two meta-analyses were published showing conflicting results [48,49]. Furthermore, methodological limitations hampered definitive conclusions from these two publications [18]. Interestingly, the FIRE-3 trial is a first-line setting study which compared FOLFIRI plus bevacizumab versus FOLFIRI plus cetuximab [50]. Among *BRAF^V600E^* mutant mCRC enrolled in this trial (*N* = 48; 14%), cetuximab led to a higher ORR but no difference in terms of PFS and OS were captured between the two arms [50]. In conclusion, latest NCCN guidelines recommend the use of anti-EGFR agents only in *BRAF* wild-type tumor, while ESMO guidelines are less restrictive [3,4]. Overall, anti-EGFR drugs represent a weak option for treatment of *BRAF^V600E^* mutant mCRC patients in the first line and even more in further lines of therapy (Figure 2).

### 2.3. Triplet Cytotoxic Combination Plus Biological Agent

In all mCRC patients, an intensive chemotherapy regimen of FOLFOXIRI plus a bevacizumab can be considered in the first-line setting [18]. In particular, this regimen is currently recommended by clinical guidelines for *BRAF^V600E^* mutant mCRC fit (meaning ECOG performance status 0 or 1) patients [3,4]. Initially, a phase II trial with FOLFOXIRI plus bevacizumab specifically designed for *BRAF^V600E^* mutant mCRC showed promising results in terms of median PFS and OS [51]. Following, in a subgroup analysis of the phase III TRIBE study, *BRAF^V600E^* mutant mCRC patients appeared to benefit more from triplet combination plus bevacizumab if compared to FOLFIRI plus bevacizumab, even if statistical significance was not reached (Table S1) [28]. In contrast, the TRIBE 2 study did not confirmed the advantage of FOLFOXIRI plus bevacizumab versus the doublet regimens plus bevacizumab in the *BRAF^V600E^* mutant mCRC patients [52]. This has been recently confirmed by a meta-analysis from the same group, demonstrating no benefit from FOLFOXIRI plus bevacizumab if compared to standard doublet cytotoxic combinations [53]. These data relight the debate on current clinical guidelines recommendation, making FOLFOXIRI plus bevacizumab no longer the treatment of choice in first line for *BRAF^V600E^* mutant mCRC patients [3,4,53]. In addition to FOLFOXIRI plus bevacizumab, in the randomized phase II VOLFI trial FOLFOXIRI plus panitumumab has also been studied in first-line, showing a high ORR improvement (85% versus 22%) in *BRAF^V600E^* mutant mCRC [54], requiring confirmation in larger studies and possibly leading to reconsider the role anti-EGFR in this setting.

### 2.4. BRAF-Targeted Combinations

In *BRAF^V600E^* mutant melanoma BRAF-inhibition led to dramatic results both in the metastatic and adjuvant settings [55,56,57]. In mCRC, initial studies of monotherapy with BRAF-inhibitors provided poor outcomes, with fewer than 10% responders and poor PFS [22,23]. Subsequently, preclinical studies allowed to shed light on mechanisms of primary resistance to BRAF blockade in this tumor: differently from melanoma, in CRC anti-BRAF monotherapy induces a feedback activation of EGFR that re-activate the oncogenic pathway providing pharmacological escape [27] (Figure 1). As a consequence, multiple studies combined EGFR and BRAF inhibitors in *BRAF^V600E^* mutant mCRC, demonstrating improved results [24,58,59]. Recently, the BEACON phase III trial compared the combination of the newer anti-BRAF agent encorafenib plus the MEK inhibitor binimetinib and cetuximab versus encorafenib and cetuximab versus FOLFIRI or irinotecan plus cetuximab after failure of first-line therapy [25]. In this study, both the triplet and the doublet combinations were superior to control arm obtaining a median OS of nine and 8.4 months respectively, compared to 5.4 months in the control arm [25]. Objective responses were 29% with the triplet, 23% with the doublet and 2% with the control arm [25]. Severe toxicities of grade 3 and higher were reported in 58% of patients in the triplet arm, 50% in the doublet and 61% in control arm [25]. Based on these data, FDA and EMA recently approved the doublet combination of cetuximab plus encorafenib after failure of a first-line treatment for *BRAF^V600E^* mutant mCRC patients (Table S1) [60,61]. This study has been criticized in two aspects. First, the percentage of MSI CRC patients enrolled was lower than 10% which appears lower than expected among *BRAF^V600E^* mutant mCRC [15,16,62]. Second, the control arm has been questioned since the use of a regimen including an anti-EGFR in the second line setting is of very limited efficacy [44,45]. Based on the BEACON results, the active but not recruiting phase II ANCHOR-CRC trial is going to explore the role of the triplet combination in first-line setting [63]. Initial results of the triplet combination in the first-line setting were recently presented and demonstrated a 50% ORR and 85% DCR with a mPFS 4.9 months and a safety profile similar to the BEACON study [63]. Based on that, a comparison with standard chemotherapy in the upfront setting is awaited with great interest. In addition to these combinations, preclinical data described an increased PI3K/AKT pathway activation as a possible mechanism of resistance to BRAF-targeted monotherapy (Figure 1), thus cetuximab and encorafenib has been compared to the triplet cetuximab, encorafenib plus alpelisib [64]. ORR was 19% and 18% while median PFS was 3.7 and 4.2 months for the doublet and triplet, respectively [64]. Further studies are warranted to clarify the potential role of adding alpelisib to the combination of cetuximab and encorafenib. Finally, it should be considered that acquired resistance eventually takes place also in face of multiple layers of BRAF-blockade, being associated with the expansion of pre-existing minor *RAS* mutant clones [24]. In this regard, in vitro data suggest considering an upfront convergent targeting with also an ERK inhibitor to prevent resistance [24].

Overall, even if the recent approval of the combination of cetuximab and encorafenib represents a step forward for treatment of *BRAF^V600E^* mutant mCRC, it is estimated that only 60% of these patients actually reach second-line treatment due to the aggressiveness of this disease [11,12]. Because of this prognostic impact, it is therefore crucial to consider for all *BRAF^V600E^* mCRC patients early enrollment in clinical trials right from the first-line setting.

## 3. Ongoing Clinical Trials

### 3.1. Material and Methods

**Figure 3:**
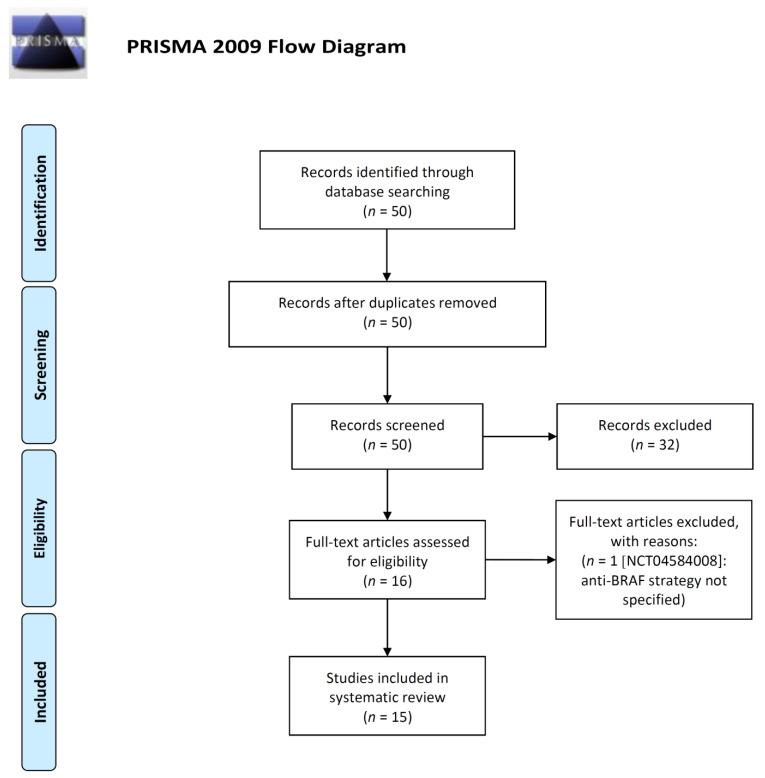
PRISMA 2009 Flow diagram representing the systematic review performed on ClinicaTrial.gov on 23 December 2020 [65]. For more information, visit www.prisma-statement.org.

An initial systematic review process was performed on 23 November and then updated throughout the revision process on 23 December 2020 with the aim to guarantee a more comprehensive and timely assessing of the panorama of strategies currently under investigations harnessing *BRAF^V600E^* mutant mCRC. We performed a systematic review of ongoing clinical trials on Clinicaltrial.gov according to PRISMA guidelines (Figure 3) [65]. The Medical Subject Headings terms used for the search in ClinicalTrials.gov were (“Recruiting or not yet recruiting” as status), (“colo-rectal cancer” as condition/disease) and (“BRAF” as other terms). The systematic review process was performed independently by two authors (G.M. and V.G). and checked by other two authors (E.B. and A.S-B.). All ongoing studies not detailing the anti-BRAF regimen under investigation were excluded.

### 3.2. Results

The treatment panorama of *BRAF^V600E^* mutant mCRC is evolving according to the results of focused clinical studies. Besides the above-mentioned trials (Table S1) [25,28,35], many others are currently ongoing to further improve prognosis of these patients. To capture the whole picture of clinical strategies specifically directed to *BRAF^V600E^* mutant mCRC, we collected data of currently ongoing clinical trials in this subset of patients. Throughout a systematic review process, we gathered 50 studies of whom 16 were assessed for eligibility and 15 included in this review (Table 1 and Figure 3). One trial (NCT04584008) was excluded since the anti-BRAF treatment strategy is not detailed. Indeed, the clinical studies identified were classified according to the treatment strategy adopted: targeting MAPK pathway (monotherapy or combinations), targeting MAPK pathway combined with cytotoxic agents, intensive cytotoxic regimens plus standard biological agents, targeted agents combined with CPIs, oxidative stress induction and cytotoxic agents combined with antiangiogenic drugs and CPIs (Table 1).

The first strategy currently under investigation to target *BRAF^V600E^* mutant mCRC includes the use of agents targeting the MAPK pathway, alone or in combination, and it is currently one of the more represented with 5 ongoing clinical trials. Three of them are phase I while two are phase II trials. One trial (NCT04294160) is evaluating multiple targeted combinations of BRAF inhibitors, ERK inhibitors, SHIP2 inhibitors or pan-RAF inhibitor (Figure 1) [66,67]. A further study (NCT03714958) is testing the option of targeting the P53 inhibitor MDM2 and MEK [68,69].

The second strategy being tested is to combine MAPK targeting agents with cytotoxic agents. We retrieved three studies currently pursuing this option. One of them is a phase II trial (NCT03727763) combines FOLFIRI with cetuximab and vemurafenib based on previous encouraging data combining irinotecan with anti-BRAF molecules [58,59]. Among them, the BREAKWATER study (NCT04607421) is the only phase III trial currently ongoing specifically designed for *BRAF^V600E^* mutant mCRC patients. Based on promising results from BEACON and ANCHOR-CRC trials [25,63], this trial is evaluating the efficacy of the combination cetuximab plus encorafenib compared to the same combination plus FOLFOX or FOLFIRI compared to physician choice. Interestingly, MSI patients are excluded unless they are ineligible to receive CPIs. Furthermore, the intensive regimen FOLFOXIRI plus bevacizumab is allowed among physician choices in the control arm.

The third strategy is represented by the upfront administration of intensive cytotoxic regimens combined with standard biological agents. Considering the high number of *BRAF^V600E^* mutant mCRC patients who will never receive a second-line treatment, the rational of this strategy is to maximize treatment outcome within the first-line setting [11,12]. TRIBE and VOLFI trials do support a potential benefit of this approach [28,54]. The AIO-KRK-0116 trial is a randomized phase II trial (NCT04034459) comparing FOLFORIXI plus cetuximab versus FOLFOXIRI plus bevacizumab. This trial is expected to provide data on intensive regimens efficacy and tolerability and to define the role of anti-EGFR compared to anti-VEGF agents on top of FOLFOXIRI.

The fourth strategy combines targeted agents with CPIs such as nivolumab or spartalizumab (PDR001). Five studies have been retrieved. Two of them are evaluating BRAF and MEK inhibitors combined with a CPI (NCT03668431 and NCT04044430) and one is testing a combination of BRAF and ERK inhibitors with spartalizumab (NCT04294160). Another one is investigating cetuximab plus encorafenib combined with nivolumab (NCT04017650), while the last one is testing a porcupine inhibitor with spartalizumab (NCT01351103). Similarly to early trials of BRAF targeting in mCRC, this strategy has been derived from melanoma [70,71]. In CRC, a positive correlation between the expression of programmed death ligand-1 (PD-L1) and the presence of *BRAF^V600E^* mutation has been shown, with also higher levels of CD8+ tumor-infiltrating lymphocytes [72]. This led to reason that *BRAF^V600E^* mutant MSS mCRC patients might benefit from a combination of targeted agents and a CPI. Interestingly, initial results obtained with the combination of dabrafenib, trametinib and spartalizumab (NCT03668431) were recently presented and demonstrated a promising 35% ORR and 75% DCR [73]. Of note, patients pretreated with CPIs or BRAF inhibitors were allowed to enter the trial but efficacy was reported lower [73]. Translational analysis of circulating tumor DNA (ctDNA) and patients-derived organoids (PDO) carried out in this trial are expected to clarify mechanisms of resistance and efficacy of this approach [73]. Further results from these studies are awaited with great interest.

The fifth strategy currently under investigation is the exploitation of the oxidative stress induced by high-dose vitamin C administration. Vitamin C has been preclinically demonstrated able to selectively kill *RAS* and *BRAF* mutant mCRC cells [74]. This killing activity is mediated by the stalling of glyceraldehyde 3-phosphate dehydrogenase, (GAPDH) which causes an energetic crisis in highly glycolytic *KRAS* and *BRAF* mutant but not in wild-type CRC cells (Figure 1) [74]. Following this study, a currently ongoing trial is investigating this strategy in *RAS* and *BRAF* mutant mCRC (NCT03146962). Recently, an enhanced activity of CPIs induced by concomitant administration of vitamin C has been reported [75]. Further clinical studies are warranted to test this combination in this subset of patients.

Finally, the sixth strategy under investigation harnessing *BRAF^V600E^* mutant mCRC is an intensive approach combining cytotoxic agents plus an antiangiogenic drug and a CPI in patients receiving first- or second-line treatment (NCT04653480). Similarly to intensive cytotoxic regimens combined with standard biological agents, this last approach aims to sooner maximize treatment outcome [11,12].

## 4. Discussion 

*BRAF^V600E^* mutant mCRC is a currently an unmet medical need requiring both preclinical and clinical research. Even if dedicated treatment options have been included in the latest clinical guidelines, prognosis of *BRAF^V600E^* mutant mCRC patients is still dismal [3,25,28]. Accordingly, many clinical trials are currently ongoing (Table 1) and given the amount of research targeting this subset of CRCs, clinical recommendations are likely to change in the future. Differently from *BRAF^V600E^* mutant mCRC, *BRAF^non-V600E^* mutant mCRC are usually left-sided, non-mucinous, MSS, without peritoneal involvement leading to a better OS, and not requiring the same treatment approach [7,8,11].

Differently from recent publications on this topic [11,18,76], in this review we focused on ongoing clinical trials with the aim to define future developments of treatment for this subset of patients [11,18,76] (Figure 3). Our search led to identification of six different treatment strategies directed against *BRAF^V600E^* mutant mCRC. Among these, the exploitation of agents targeting the MAPK pathway and intensive chemotherapy regimens appear as the most promising based on previous results derived from published clinical trials [25,28]. However, a recent meta-analysis, showing no benefit from FOLFOXIRI plus bevacizumab if compared to standard cytotoxic doublets plus bevacizumab in *BRAF^V600E^* mutant mCRC, will lead to reconsider current clinical guidelines recommendations [3,4,53]. Moreover, the combination of targeted agents plus CPIs is of great interest, particularly for MSS tumors, even if there are only initial data in mCRC [73]. Among other avenues, a provocative opportunity is represented by high-dose vitamin C, even though several issues are still to be addressed such as the right dosages and infusion scheduling.

*BRAF^V600E^* mutation is commonly recognized as a poor prognostic factor in mCRC with a median OS of less than 20 months for metastatic disease [12]. However, around 20% of patients with *BRAF^V600E^* mCRC patients survives beyond 24 months from the initial diagnosis [12,28,51,77,78]. The reason for this prognostic heterogeneity has not been identified yet. According to molecular consensus subtypes (CMS), *BRAF^V600E^* mutant mCRC are identified for the vast majority in the CMS1 subgroup while the few remaining are scattered across the other CMS subtypes [13]. However, CMS classification does not explain this prognostic heterogeneity. Barras and coworkers from a cohort of 218 *BRAF^V600E^* mutant CRC identified two subtypes of disease with different prognosis: BM1 (*BRAF* mutant 1) and BM2 (*BRAF* mutant 2) [79]. These two subgroups were characterized by substantial differences both at transcriptomic and proteomic level and they are independent from patients’ gender, sidedness, MMR status and PI3K status [79]. BM1 is less common (1/3 of cases) and is characterized by strong activation of AKT/mTOR, *KRAS*, *4EBP1* and epithelial-mesenchymal transition features [79]. On the other hand, BM2 represent most of cases and it is characterized by cell cycle deregulation, high level of CDK1 and low level of cyclin D1 [79]. Despite prognostic subdivision, this classification has no direct implication for the *BRAF^V600E^* treatment decision algorithm. In addition to molecular characterization, a retrospective platform of 395 *BRAF^V600E^* mutant mCRC led to the identification of three different prognostic subgroups based on the use of clinical data [80]. Even if this classification might have potential implication for treatment decision and for guiding translational research, its integration with molecular classification such as BM1/BM2 or CMS is warranted [80]. It should be noted that neither any molecular sub-grouping nor clinical classification has been used to date to design ongoing clinical trials against *BRAF^V600E^* mutant mCRC. A closer interaction between preclinical and clinical researchers is needed therefore to design future trials.

## 5. Conclusions

The treatment of *BRAF^V600E^* mutant mCRC has been relentlessly improving over the last decade thanks to the parallel evolution of preclinical and clinical knowledge. The advent of cancer immune therapy with CPIs has clearly changed the scenario providing striking results also in this subset of MSI tumors, although the true challenge is represented by patients harboring *BRAF^V600E^* MSS cancer. Results of currently ongoing clinical trials exploiting new strategies, such as the combination of different targeted agents and with CPIs, are awaited to further expand the spectrum of treatment for this peculiar subtype of CRC under the paradigm of precision oncology.

## Figures and Tables

**Table 1:** Interventional ongoing clinical trials targeting specifically *BRAF^V600^* mutant metastatic colorectal cancer (mCRC) retrieved through a systematic review process performed on 23 December 2020.

Strategy	Study ID Status Main Location	Ph.	Drug Schedule	Main Inclusion Criteria
*Targeting MAPK pathway (monotherapy or combinations)*	NCT04294160 Recruiting Germany	Ib	-Dabrafenib + LTT462 (ERKi) -Dabrafenib + Trametinib + LTT462 (ERKi) -Dabrafenib + LTT462 (ERKi) + LXH254 (pan-RAFi) -Dabrafenib + LTT462 (ERKi) + TNO155 (SHP2i)	-*BRAF^V600^* mutation -Site for biopsy at baseline and on treatment
	NCT03087071 Recruiting USA	II	-Panitumumab + Trametinib (*Cohort 2*)	-*KRAS*, *NRAS*, or *BRAF* mutation -Prior treatment with MEKi, ERKi or anti-EGFR not allowed
	NCT03714958 Recruiting France	I	-Trametinib + HDM201 (Mdm2i)	-*RAS* or *BRAF* mutation and *TP53* wild-type (also *BRAF* translocation are eligible)
	NCT02465060 (MATCH) Recruiting USA	II	-Dabrafenib + Trametinib	-Solid tumor with *BRAF^V600E/R/K/D^* mutation
	NCT04190628 Recruiting USA	I	-ABM-1310 (BRAFi)	-Solid tumor *BRAF^V600^* mutation -Patients with active brain metastases are eligible
*Targeting MAPK pathway combined with cytotoxic agents*	NCT03727763 (IMPROVEMENT) Recruiting China	II	-FOLFIRI + vemurafenib + cetuximab	-*BRAF^V600E^* mutation and extended *RAS* wild-type
	NCT02857270 Recruiting USA	Ib	-LY3214996 (ERK1/2i) ± other agents (*Part E*)	-*BRAF^V600E^* mutation
	NCT04607421 (BREAKWATER) Not yet recruiting	III	-Encorafenib + Cetuximab ± FOLFOX/FOLFIRI vs. FOLFOX/FOLFIRI/FOLFOXIRI/CAPOX ± bevacizumab	-*BRAF^V600E^* mutation -1st line treatment -MSI is an exclusion criteria unless the patient is not eligible to CPIs
*Intensive cytotoxic regimens plus standard biological agents*	NCT04034459 (AIO-KRK-0116) Recruiting Germany	II	-FOLFOXIRI + cetuximab -FOLFOXIRI + bevacizumab	-*BRAF^V600E^* mutant and pan-*RAS* wild-type -1st line treatment
*Targeted agents combined with checkpoint inhibitors*	NCT03668431 Recruiting USA	II	-Dabrafenib + Trametinib + Spartalizumab (PDR001)	-*BRAF^V600E^* mutation and pan-*RAS* wild-type -Any line -prior anti-EGFR, BRAFi or MEKi, or prior CPIs allowed
	NCT04294160 Recruiting Germany	Ib	-Dabrafenib + LTT462 (ERKi) + Spartalizumab (PDR001)	-*BRAF^V600E^* mutation -Site for biopsy at baseline and on treatment
	NCT01351103 Recruiting USA	I	-LGK974 (porcupine inhibitor) ± Spartalizumab (PDR001)	-BRAF mutant colorectal cancer ± RNF43 mutation and/or RSPO fusion
	NCT04017650 Recruiting USA	I/II	-Cetuximab + Encorafenib + Nivolumab	-*BRAF^V600E^* mutation MSS -Prior BRAFi, MEKi, ERKi, anti-EGFR and CPIs not allowed
	NCT04044430 Recruiting USA	I/II	-Encorafenib + Binimetinib + Nivolumab	-*BRAF^V600E^* mutation MSS -prior anti-EGFR, BRAFi or MEKi, or prior CPIs not allowed
*Oxidative stress induction*	NCT03146962 Recruiting USA	II	-Vitamin C	-*RAS* (e.g. *KRAS* or *NRAS*) or *BRAF* mutation
*Cytotoxic agents combined with antiangiogenic drugs and checkpoint inhibitors*	NCT04653480 Recruiting China	II	-Oxaliplatin or irinotecan cytotoxic regimens + Surufatinib (anti-VEGFR and FGFR) + Toripalimab (anti-PD-1)	-RAS or BRAF mutation MSS -less than 2 previous systemic line of treatment

Legend: Ph. = phase; i = inhibitor; CPI = checkpoint inhibitors.

## Supplementary Materials

The following are available online at https://www.mdpi.com/2072-6694/13/1/137/s1, Table S1: Main completed clinical trials which demonstrated to improve clinical outcome in BRAFV600E mutant metastatic colorectal cancer (mCRC) if compared to standard doublet plus anti-VEGF or anti-EGFR agents and supporting the current recommendation by NCCN and ESMO clinical guidelines.
