# Supplementary material for: The Evolutionary Landscape of Treatment for BRAFV600E Mutant Metastatic Colorectal Cancer
Source: Cancers (Basel). 2021 Jan 4;13(1):137. doi: 10.3390/cancers13010137 (PMC7795863; doi:10.3390/cancers13010137)
Supplement: Supplementary file 1 [file cancers-13-00137-s001.pdf]

The Evolutionary Landscape of Treatment for *BRAF*^{V600E} Mutant Metastatic Colorectal Cancer

Gianluca Mauri, Erica Bonazzina, Alessio Amatu, Federica Tosi, Katia Bencardino, Viviana Gori, Daniela Massihnia, Tiziana Cipani, Francesco Spina, Silvia Ghezzi, Salvatore Siena, and Andrea Sartore-Bianchi

Table S1. Main completed clinical trials which demonstrated to improve clinical outcome in *BRAF*^{V600E} mutant metastatic colorectal cancer (mCRC) if compared to standard doublet plus anti-VEGF or anti-EGFR agents and supporting the current recommendation by NCCN and ESMO clinical guidelines.

Therapeutic strategy	Regimen	Phase	Line of treatment	Patients * (N)	ORR (%)	mPFS (months)	Reference
Immunotherapy (MSI only)	Pembrolizumab (*KEYNOTE-177*)	III	1st	34	43.8**	16.5**	35
Targeting MAPK pathway	Encorafenib + cetuximab (*BEACON*)	III	2nd or 3rd	220	20	4.2	25
Intensive cytotoxic regimen	FOLFOXIRI + bevacizumab (*TRIBE*)	III	1st	16	56	7.6	28

<u>Keys</u>: *=number of patients enrolled in the trial and treated with the regimen described in each line. **=data regarding the entire cohort of metastatic colorectal cancer patients treated with pembrolizumab (N=153) and not limited to those affected by *BRAF*^{V600E} mutant mCRC. <u>Legend</u>: ORR=overall response rate; N=number of patients included in the trial affected by *BRAF*^{V600E} mutant colorectal cancer; mPFS=median progression-free survival.
